# Efficacy of Sacrospinous Fixation or Uterosacral Ligament Suspension for Pelvic Organ Prolapse in Stages III and IV: Randomized Clinical Trial

**DOI:** 10.1055/s-0043-1772592

**Published:** 2023-11-09

**Authors:** Sérgio Brasileiro Martins, Rodrigo de Aquino Castro, Claudia Cristina Takano, Gisele Vissoci Marquini, Leticia Maria de Oliveira, Paulo Cezar Feldner Martins Junior, Márcia Maria Dias, Manoel João Batista Castello Girão, Marair Gracio Ferreira Sartori

**Affiliations:** 1 Department of Gynecology, Escola Paulista de Medicina, Universidade Federal de São Paulo, São Paulo, SP, Brazil

**Keywords:** pelvic organ prolapse, pelvic floor disorders, reconstructive surgical procedures, patient health questionnaire, patient-reported outcome measures, prolapso de órgão pélvico, distúrbios do assoalho pélvico, procedimentos cirúrgicos reconstrutivos, questionário de saúde da paciente, medidas de resultados relatadas pela paciente

## Abstract

**Objective** To evaluate the efficacy and outcomes of the surgical treatment for pelvic organ prolapse (POP) in stages III and IV by sacrospinous ligament fixation (SSLF) or uterosacral ligament suspension (USLS) by comparing anatomical and subjective cure rates and quality-of-life parameters (through the version validated for the Portuguese language of the Prolapse Quality of Life [P-QoL] questionnaire) under two definitions: genital prolapse Ba, Bp, and C < −1 (stage I) and Ba, Bp, and C ≤ 0 (stage II).

**Materials and Methods** After we obtained approval from the Ethics Committee (under CAAE 0833/06) and registered the study in ClinicalTrials.gov (NCT 01347021), 51 patients were randomized into two groups: the USLS group (N = 26) and the SSLF group (N = 25), with follow-up 6 and 12 months after the procedures.

**Results** There was a significant improvement in the P-QoL score and anatomical measurements of all compartments in both groups after 12 months ( *p* < 0.001). The anatomical cure rates in the USLS and SSLF groups, considering stage 1, were of 34.6% and 40% (anterior) respectively; of 100% both for groups (apical); and of 73.1% and 92% (posterior) respectively. The rates of adverse outcomes were of 42% (N = 11) and 36% (N = 11) for the USLS and SSLF groups respectively ( *p* = 0.654), and those outcomes were excessive bleeding, bladder perforation (intraoperative) or gluteal pain, and urinary infection (postoperative), among others, without differences between the groups.

**Conclusion** High cure rates in all compartments were observed according to the anatomical criterion (stage I), without differences in P-QoL scores and complications either with USLS or SSLF for the surgical treatment of accentuated POP.

## Resumo

**Objetivo** Avaliar a eficácia e os resultados do tratamento cirúrgico para prolapso de órgãos pélvicos (POP) nos estágios III e IV, por meio da técnica de fixação do ligamento sacroespinal (FLSE) ou suspensão do ligamento útero-sacro (SLUS), ao comparar os índices de cura anatômicos, subjetivos, e os parâmetros de qualidade de vida (por meio do questionário Prolapse Quality of Life [P-QoL] validado para a língua portuguesa) sob duas definições: prolapso genital Ba, Bp e C < −1 (estágio I) e Ba, Bp e C ≤ 0 (estágio II).

**Materiais e Métodos** Após aprovação do Comitê de Ética (CAAE 0833/06) e registro no ClinicalTrials.gov (NCT 01347021), 51 pacientes foram randomizadas em dois grupos: grupo SLUS (N = 26) e (2) grupo FLSE (N = 25), com seguimento de 6 e 12 meses.

**Resultados** Houve melhora significativa nas pontuações no P-QoL e nas medidas anatômicas de todos os compartimentos em ambos os grupos após 12 meses ( *p* < 0,001). As taxas de cura anatômica nos grupos SLUS e FLSE , considerando o estágio 1, foram de 34,6% e 40% (anterior), respectivamente; de 100% em ambos os grupos (apical); e de 73,1% e 92% (posterior), respectivamente. As taxas de resultados adversos foram de 42% (N = 11) e 36% (N = 11), respectivamente, nos grupos SLUS e FLSE ( *p* = 0,654), e elas foram sangramento excessivo, perfuração da bexiga (intraoperatória) ou dor glútea, e infecção urinária (pós-operatória), entre outras, sem diferenças entre os grupos.

**Conclusão** Altas taxas de cura em todos os compartimentos foram observadas segundo critério anatômico (estágio I), sem diferença quanto às pontuações no P-QoL e às complicações tanto com SLUS quanto com FLSE para o tratamento cirúrgico de POP acentuado.

## Introduction

In high-income countries, individuals are growing ever older, and the need for pelvic organ prolapse (POP) treatment is anticipated to increase in the coming decades. 1 The treatment of the apical compartment is critical to the successful repair of severe POP. The two most commonly used techniques for apical corrections, through vaginal procedures, are uterosacral ligament suspension (USLS) or sacrospinous ligament fixation (SSLF). 2 3 4 Close to the ischial spines, USLS is an effective intraperitoneal procedure to restore apical support in 98% of women. 5 The second technique is a widely used extraperitoneal procedure with subjective cure rates ranging from 70% to 98%, and objective cure rates ranging from 67% to 97%. 6

In an attempt to improve the anatomical result of pelvic reconstruction surgeries with native tissues, the use of meshes was propagated with the intention of replacing the injured native tissues, but numerous complications and high rates of reoperations due to exposure, pain, and dyspareunia have been observed. 2 3 4 7 Because of warnings about the adverse effects of surgical correction with polypropylene meshes, 7 efforts to identify the ideal technique to correct the apical effect by the vaginal access have been undertaken. 7

Thus, medical societies specializing in such fields have recommended that meshes be used sparingly, with restrictions or not at all. It is possible that researchers in countries such as the United States, Australia, and the United Kingdom have stopped their studies on this topic because of mesh scrutiny. 1 7 Therefore, the objective of the present study was to compare the success rates and outcomes of USLS and SSLF in the surgical treatment of advanced apical POP (stages III and IV) under the subjective (Ba, Bp and C < −1) and anatomical (Ba, Bp and C ≤ 0) cure criteria of the Pelvic Organ Prolapse Quantification (POP-Q) System. 8

## Materials and Methods

The present prospective and randomized trial was performed at Universidade Federal de São Paulo, Brazil, and it was approved by the Research Ethics Committee of said institution (under CAAE 0833/06; CEP 0833/06 [attached document]) and registered on Clinicaltrials.gov (NCT 01347021). The inclusion criteria were patients with apical POP in stages III or IV, aged between 50 and 90 years, who voluntarily agreed to participate and signed the informed consent form. The exclusion criteria were clinico-surgical contraindications (severe comorbidities), apical POP in stages I and II; previous pelvic radiotherapy or thromboembolic disorders; hormone therapy; endometrial hyperplasia or high-grade squamous intraepithelial lesions of the cervix, vagina, or vulva, or untreated genitourinary infection.

**Fig. 1:**
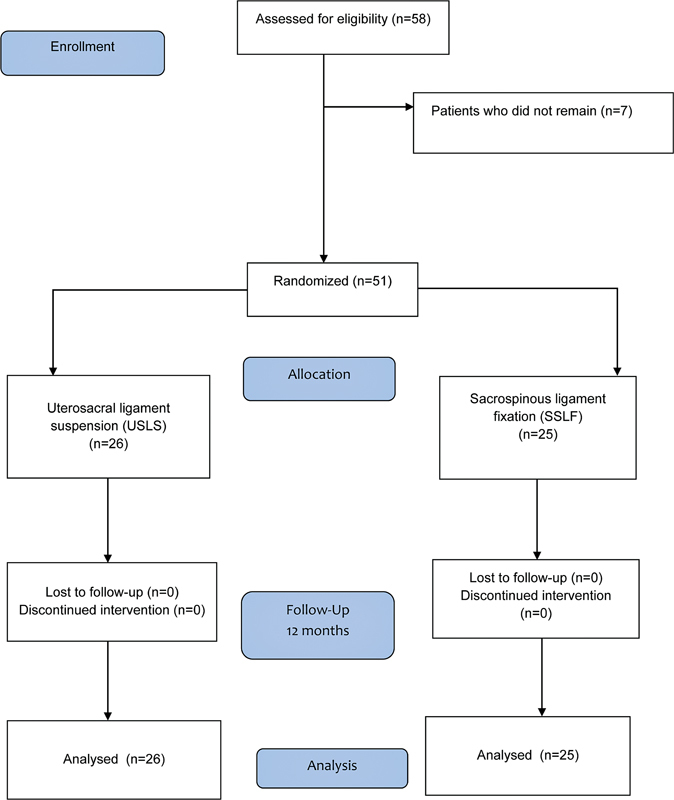
Flow diagram of the present study.

Standard history-taking was performed, as well as a physical examination in the supine and standing positions to stage the POP through the POP-Q according to the recommendations of the International Continence Society (ICS), 8 9 followed by reduction of the prolapse using gauze and a Cheron dressing forceps. The stress test was performed with and without the prolapse reduction to diagnose occult stress urinary incontinence (SUI). If involuntary leakage of urine was observed, a urodynamic study was performed to include the correction of the SUI during the surgical approach. All clinical evaluations were performed by the authors SBM and CCT through sequential randomization (performed by LMO and MMD) and allocation of the sample into two groups. The double-blinded randomization criterion was not applied because the procedures would have had to be explained to the patient and performed by the authors (SBM, CCT, LMO and MMD) in a technically-feasible manner with the scientific rigor of sequential randomization. However, the statistician and the preoperative evaluator were blinded because they did not know to which group the patient would be assigned. The random allocation was sequential and performed 1:1 by drawing lots to avoid possible selection biases, and, in the postoperative evaluation, there was no blinding. The authors state that this does not compromise the outcomes, since the postoperative evaluator was blinded to the other randomization processes. The initial sample consisted of 58 patients with stage III and IV apical POP (according to the POP-Q). Of these, 7 were excluded (leaving 51 randomized patients) ( Fig. 1 ) because they presented stage II apical prolapse. Vaginal hysterectomy (VH) was performed in all patients, and correction of SUI with a retropubic midurethral sling when indicated, followed by cystoscopy to assess ureteral integrity and correction of site-specific apical defects through USLS or SSLF. Nine SUI corrections were performed in the USLS group and seven in the SSLF group. All patients underwent posterior colporrhaphy and perineorrhaphy, without the need for a description of the technique, as this was not the technique under comparison in the study hypothesis. The analyses were performed using the intention-to-treat (ITT) principle. In the USLS group, the uterosacral ligaments were identified, seized with an Allis forceps ∼ 1 cm medial and posterior to the ischial spine, and repaired, followed by a procedure to correct site-specific defects (in the anterior compartment). After that, the sutures of the uterosacral ligament were passed in the vaginal apex followed by anterior, posterior and perineorrhaphy or colporrhaphy. 10 11 In the SSFL group, correction of site-specific defects (in the anterior compartment) was performed by means of a longitudinal incision of the posterior vaginal wall up to 2 cm from the vaginal apex. Correction of the site-specific defects of the posterior vaginal wall and enterocele was performed, and the knots of polypropylene threads (number 0) were tied, leading the vaginal apex toward the sacrospinous ligament, avoiding excessive traction. 10 11 All patients received intravenous antibiotic therapy (cephalothin and metronidazole) intraoperatively. Meshes were not used to correct pelvic floor defects. Hospital discharge occurred at least 48 hours after surgery, provided the patients were clinically well.

Quality of life was assessed preoperatively, 6, and 12 months after the intervention through the version validated for the Portuguese language of the Prolapse Quality of Life (P-QoL) questionnaire, 12 which determined the criterion of subjective cure. This questionnaire contains 20 questions in 9 domains: general health perception; prolapse impact; role limitations; physical limitations; social limitations; personal relationships; emotions; sleep/energy; and severity measures. 12

Anatomical success was evaluated by the positions of the vaginal apex, anterior and posterior compartments after 6 and 12 months of follow-up in the two groups. Data from 12 months of follow-up were used to assess both quality of life and the anatomical outcomes, because of the greater practical applicability of the outcomes. However, the authors consider the follow-up of patients to assess long-term efficacy, which was not the objective of the present study. For the assessment of cure ranges, two specific clinical criteria were used: according to the first criterion, those with the highest prolapse point lower than −1 according to the POP-Q were considered cured (stage I). Then, in the second criterion, patients were regrouped and considered cured when the point of greatest prolapse was ≤ 0, (stage II). 8

The duration of the surgery (time from the first incision to the completion of the suture) and blood loss (hemoglobin and hematocrit levels) were also analyzed pre- and postoperatively. Complications were described according to the terminology of the International Urogynecological Association (IUGA) and ICS. 13

The sample size (N) was calculated based on the primary objective of the study. According to the literature review, the authors estimated a minimum of 20 patients in each group, with a difference of 25% between them. The level of rejection of the null hypothesis was set at 0.05 or 5% (α ≤ 0,05) and the power of the sample, at 80%. 14 The statistical analyzes were performed using the IBM SPSS Statistics for Windows (IBM Corp., Armonk, NY, United States), version 19.0, and the R software (R Foundation for Statistical Computing, Vienna, Austria), version 2.11.1. Quantitative (numerical) variables were calculated as mean, median, minimum and maximum, and standard deviation values. The qualitative (categorical) variables were analyzed by calculating their absolute and relative frequencies (percentage). The Student *t* - and Mann-Whitney tests were used to compare the continuous variables, and the Pearson Chi-squared with the Fisher exact tests, to compare the categorical variables between groups. Analysis of variance (ANOVA) was performed to compare the POP and the P-QoL score between the 2 groups before and 12 months after surgery. The significance level was set at 0.05.

## Results

The two groups were homogeneous in terms of age, age at the onset of menopause, number of pregnancies, vaginal or cesarean deliveries, body mass index (BMI), race, smoking status, systemic arterial hypertension, diabetes mellitus, initial stage, previous gynecological surgeries, presence of SUI or occult SUI ( Table 1 ). After 12 months of follow-up, a significant improvement was observed in all anatomical points after the surgeries in both groups ( Table 2 ).

**Table 1:** Preoperative characteristics of the patients

	USLS	SSFL	*p*
**Age (years)** (min–max) ± SD	68.8 (50–88) ± 10.3	66.6 (53–80) ± 6.9	0.374ª
**Parity** (min–max) ± SD	5,0 (1–14) ± 3.4	5,4 (0–15) ± 4.0	0.648 ^a^
**Vaginal deliveries** (min–max) ± SD	4,1 (0–14) ± 3.6	4,2 (0–12) ± 3.5	0.864 ^b^
** BMI (Kg/m ^2^ ) ** (min–max) ± SD	26,2 (17.7–38.9) ± 4.7	25,8 (19.4–33.9) ± 3.2	0.760 ^a^
**Race**			0.068 ^c^
White	18 (69.2%)	19 (76%)	
Non-white	8 (31.8%)	6 (24%)	
**POP-Q stage**			0.312 ^d^
III	13 (50%)	9 (36%)	
IV	13 (50%)	16 (64%)	
**Previous surgeries**	5 (19.2%)	7 (28%)	0.460 ^d^
SUI	15 (57.8%)	16 (64%)	0.645 ^d^
Overactive bladder	21 (80.8%)	16 (64%)	0.180 ^d^
Occult SUI	3 (11.5%)	4 (12%)	> 0.999 ^c^

Abbreviations: BMI, Body Mass Index; min, minimum; max, maximum; POP-Q, Pelvic Organ Prolapse Quantification System; SD, standard deviation; SSLF, sacrospinous ligament fixation; SUI, stress urinary incontinence; USLS, uterosacral ligament suspension.

Notes: ^a^ Student *t* -test for independent samples; ^b^ Mann-Whitney test; ^c^ Fisher exact test or its extension; ^d^ Pearson Chi-squared test.

**Table 2:** Position of the anatomical points (POP-Q) pre- and postoperatively after 12 months in both groups

Points	USLS		SSFL		*p*
	Mean ± SD	Min/Max	Mean ± SD	Min/Max	
Aa					
Preop	2.77 ± 0,82	(0/3)	2.48 ± 1.23	(−1/3)	0.827
Postop	−1.15 ± 1.29	(−3/1)	−1.00 ± 1.41	(−3/1)	0.778
*p* ^a^	< 0.001		< 0.001		
Ba					
Preop	4.81 ± 1.44	(1/7)	4.76 ± 2.01	(0/8)	> 0.999
Postop	−1.00 ± 1.26	(−3/1)	−0.80 ± 1.58	(−3/2)	0.791
*p* ^a^	< 0.001		< 0.001		
C					
Preop	5.73 ± 1.54	(3/8)	6.56 ± 1.69	(4/10)	0.481
Postop	−5.46 ± 1.36	(−8/−2)	−5.72 ± 1.28	(−8/−3)	0.324
*p* ^a^	< 0.001		< 0.001		
GH					
Preop	5.15 ± 0.97	(3/6)	4.80 ± 1.12	(3/8)	0.487
Postop	3.31 ± 0.68	(2/5)	3.44 ± 0.71	(2/5)	0.594
*p* ^a^	< 0.001		< 0.001		
PB					
Preop	2.73 ± 0.67	(2/4)	3.04 ± 1.14	(2/7)	0.515
Postop	3.77 ± 0.65	(3/5)	3.80 ± 0.65	(3/5)	0.388
*p* ^a^	< 0.001		< 0.001		
TVL					
Preop	8.38 ± 0.70	(7/10)	8.36 ± 0.76	(7/10)	> 0.999
Postop	7.04 ± 1.00	(5/9)	6.92 ± 1.38	(4/9)	0.951
*p* ^a^	< 0.001		< 0.001		
Ap					
Preop	0.96 ± 1.87	(−2/3)	1.56 ± 1.85	(−3/3)	0.481
Postop	−2.19 ± 1.10	(−3/1)	−2.60 ± 0.87	(−3/0)	0.105
*p* ^a^	< 0.001		< 0.001		
Bp					0.141
Preop	2.08 ± 2.86	(−2/6)	3.36 ± 2.45	(−3/8)	
Postop	−1.92 ± 1.41	(−3/2)	−2.48 ± 1.33	(−3/3)	0.043*
*p* ^a^	< 0.001		< 0.001		

Abbreviations: GH, Genital Hiatus; Min, minimum; Max, maximum; PB, Perineal Body; POP-Q, Pelvic Organ Prolapse Quantification System; Postop, postoperatively; Preop, preoperatively; SD, Standard Deviation; SSLF, sacrospinous ligament fixation; TVL, Total Vaginal Length; USLS, uterosacral ligament suspension.

Notes: Values expressed in centimeters (mean ± standard deviation, and minimum and maximum values); ^a^ Analysis of variance (ANOVA).

In the evaluation of the compartments, separately, after 12 months of follow-up, with the adoption of anatomical healing patterns of points in a position lower than −1, anatomical cure rates of 34.6% and 40% (for the USLS and SSLF groups respectively) in the anterior compartment and of 100% for both groups in the apical compartment were observed. In the posterior compartment, anatomical healing rates of 73.1% and 92% were observed in the USLS and SSLF groups respectively, with a significant improvement in the posterior compartment (Bp) favorable to the SSLF group ( *p* = 0.043) ( Tables 2 and 3 ).

**Table 3:** Cure rate using two different criteria of anatomical and functional cure: POP-Q < −1 or POP-Q ≤ 0

POP-Q	USLS ( *N* = 26) N (%)	SSLF ( *N* = 25) N (%)	*p*
Ba < −1	9 (34.6%)	10 (40.0%)	0.691 ^a^
Ba ≤ 0	23 (88.4%)	21 (84.0%)	0.703 ^a^
C < −1	26 (100.0%)	25 (100.0%)	*
C ≤ 0	26 (100.0%)	25 (100.0%)	*
Bp < 1	19 (73.1%)	23 (92.0%)	0.140 ^b^
Bp ≤ 0	23 (88.4%)	24 (96.0%)	0.610 ^b^

Abbreviations: POP-Q, Pelvic Organ Prolapse Quantification System; SSLF, sacrospinous ligament fixation; USLS, uterosacral ligament suspension.

Notes: ^a^ Pearson Chi-Squared test; ^b^ Fisher exact test; *Impossibility of performing a statistical analysis for total cure in both groups.

On the other hand, when adopting the presence of prolapse up to the hymenal caruncle as a cure criterion, that is, Ba, Bp or C ≤ 0, we observed cure rates of 88.4% and 84% (for the USLS and SSLF groups respectively) in the anterior compartment, of 88.4% (USLS group) and 96% (SSLF group) in the posterior compartment, and of 100% (both groups) in the apical compartment, with no statistical difference between techniques ( Table 3 ). Therefore, when analyzing the anatomical measurements of the compartments, there was a favorable statistical difference in the SSLF group (posterior compartment), without statistical difference between the groups when analyzing the cure rates. Using the P-QoL, we observed that, after 12 months of follow-up, both procedures were efficient, with a significant improvement in scores in the nine domains evaluated regarding the postoperative and preoperative periods. There were no significant differences when comparing both groups after 12 months of follow-up ( Table 4 ).

**Table 4:** Preoperative and postoperative P-QOL scores of women who underwent fixation of the vaginal vault through SSLF USLS

	USLS	SSLF	* p ^a^*
	Mean ± SD	Mean ± SD	
General health perception			0.970 0.514
Preop	49.0 ± 26.9	52.0 ± 24.9	
Postop	31.7 ± 24.0	22.0 ± 18.1	
* p ^b^*	< 0.001	< 0.001	
Prolapse impact			0.994 0.739
Preop	74.3 ± 36.8	76.3 ± 29.6	
Postop	8.9 ± 27.5	1.3 ± 6.6	
* p ^b^*	< 0.001	< 0.001	
Role limitations			0.555
Preop	58.9 ± 38.9	47.4 ± 40.1	
Postop	7.6 ± 27.1	0.6 ± 3.3	0.171
* p ^b^*	< 0.001	< 0.001	
Physical limitations			0.444 0.297
Preop Postop	60.9 ± 39.4 5.1 ± 20.4	48.0 ± 42.0 1.3 ± 6.6	
* p ^b^*	< 0.001	< 0.001	
Social limitations			0.771
Preop	44.8 ± 39.8	36.8 ± 38.7	
Postop	0.9 ± 4.5	0.89 ± 4.4	0.204
* p ^b^*	< 0.001	< 0.001	
Personal relationships			0.463
Preop	14.1 ± 30.4	24.0 ± 36.3	
Postop	1.2 ± 6.5	1.3 ± 6.6	0.371
* p ^b^*	< 0.001	< 0.001	
Emotions			
Preop	62.8 ± 40.9	64.4 ± 37.9	> 0.999
Postop	4.7 ± 19.6	1.3 ± 22.2	0.799
* p ^b^*	< 0.001	< 0.001	
Sleep/Energy			0.295
Preop	39.1 ± 34.2	27.1 ± 28.5	
Postop	5.7 ± 15.5	2.6 ± 10.4	
* p ^b^*	< 0.001	< 0.001	0.146
Severity measures			0.850
Preop	43.2 ± 32.4	48.3 ± 31.5	
Postop	1.9 ± 1.3	1.3 ± 5.2	0.493
* p ^b^*	< 0.001	< 0.001	

Abbreviations: P-QoL, Prolapse Quality of Life questionnaire; Preop, preoperative period; Postop, postoperative period; SD, standard deviation; SSLF, sacrospinous ligament fixation; USLS, uterosacral ligament suspension.

Note: ^a,b^ Analysis of variance (ANOVA).

The mean duration of the surgery was of 137.6 (range: 80 to 190) minutes in the USLS group, and of 146.9 (range: 80 to 215) minutes in the SSFL group, with no difference between them ( *p* = 0.299) ( Table 5 ). There was no statistical differences between the groups regarding the minimal incidence of intraoperative or postoperative complications ( Table 6 ). Subjective cure was determined by the P-QoL, and there was no difference between the groups.

**Table 5:** Comparison of perioperative results between the USLS and SSFL groups

Variables		USLS Group		SSFL Group		*p*
		Mean ± SD	Min-max	Mean ± SD	Min-max	
Operative Time (minutes)		137.6 ± 29.5	80–190	133.5 ± 33.7	80–215	0.299
Hemoglobin (g/dL)	Preop	13.1 ± 1.1	11.0–15.7	13.1 ± 1.1	10.7–15.4	0.482
	Postop	10.8 ± 1.2	8.6–13.5	11.2 ± 1.6	8.3–14.1	0.448
Hematocrit (%)	Preop	39.1 ± 3.4	34.0–47.4	39.1 ± 3.1	33.6–47.0	0.571
	Postop	32.3 ± 3.7	26.0–40.0	33.4 ± 4.7	24.7–42.6	0.415
Hospital stay (days)		2.3 ± 0.8	2.0–5.0	2.2 ± 0.8	1.0–5.0	0.559

Abbreviations: Min, minimum; max, maximum; Preop, preoperative period; Postop, postoperative period; SD, standard deviation; SSFL, sacrospinous ligament fixation; USLS, uterosacral ligament suspension.

Notes: Values expressed as mean ± standard deviation; hemoglobin and hematocrit levels collected 24 hours after surgery through analysis of variance (ANOVA) with parametric repeated measures.

**Table 6:** Total number of complications in both groups

	USLS	SSLF	* p ^a^*
**Intraoperative**			
Excessive bleeding	3	1	–
Transfusion	–	–	–
Bladder perforation	–	–	–
**Postoperative**			
Gluteal pain	–	5	–
Infection	3	1	–
De novo overactive bladder	1	–	–
Thigh paresthesia	1	–	–
Urinary infection	2	3	–
Dyspareunia	1	–	–
Rectal injury (47 ^th^ postoperative day) ^b^	–	1	–
**Total**	11	11	0.654 ^a^

Abbreviations: SSLF, sacrospinous ligament fixation; USLS, uterosacral ligament suspension.

Notes: ^a^ Pearson Chi-squared test; ^b^ one patient in the SSFL group had an acute abdomen on the 47th postoperative day, with a diagnosis of a perforation lesion measuring ∼ 5 cm, located in the middle rectum. The patient underwent exploratory laparotomy with resection of the lesion and colostomy with reconstruction of the intestinal transit in the second stage. The anatomopathological study showed circulatory disturbance, probably due to ischemic injury.

## Discussion

The present study evaluated the two most commonly performed surgical correction techniques, one of which is the reconstruction of the pelvic anatomy using native tissues, and the degree of objective (anatomical) and subjective (functional) success in achieving satisfactory results with respect to the most current recommendations on the surgical treatment of POP. 2 3 4 9 15 16

Systematic reviews, randomized trials and medical societies found no evidence to support the use of meshes to the detriment of native tissues. 2 3 4 15 16 17 Since 2011, 7 many transvaginal mesh products have been removed from the market after the Food and Drug Administration (FDA) announcement that identified serious safety, effectiveness concerns, and complications with the use of transvaginal mesh to treat POP. The outcomes of the present study corroborate global analyzes and recommendations on the surgical treatment for POP. Fortunately, VH and vaginal apex repair to the uterosacral or sacrospinous ligaments (which are relatively low-risk surgeries) are effective treatments for most women with apical POP, according to several authors and supported by renowned medical societies in the field, without the use of synthetic mesh. 2 3 4 9 15 16

Studies such as the present, which prove the effectiveness of surgical treatment with the application of traditional techniques in urogynecology, such as fixation of the vaginal vault to the sacrospinous ligament or suspension of the uterosacral ligament to correct even advanced apical prolapses, reinforce the prioritization of the choice and reproducibility of the classic technique in urogynecological surgery for POP. 17 18 19 20 Thus, currently there is no indication for the use of screens. 17 In a systematic review published in 2016, Maher et al. 17 found no evidence to support the use of meshes to the detriment of native tissues, which is in line with the results of the present study. The practical applicability of these results enables the advancement and dissemination of knowledge regarding surgical techniques in traditional urogynecology, without detriment to technologies, but with the deserved reservations. 18 19 20

Regarding the cure criteria, there is no consensus in the scientific community on success and failure in POP correction surgery. Barber et al. 8 (2009), after a 2-year follow-up of 322 patients in the Colpopexy and Urinary Reduction Efforts (CARE) study, applied 18 different definitions of success after surgery for the correction of POP in stages II to IV, and the treatment success rate varied widely depending on the definition used (range: 19.2% to 97.2%).

In that context, the POP-Q enables more accurate descriptions of the POP stages for diagnosis and follow-up. Despite the fact that most patients are classified as stage II postoperatively, with a surgical outcome between the +1 and −1 range of the hymenal ring, they generally remain asymptomatic. Therefore, what would configure an anatomical failure (stage II according to the POP-Q) is classified as a cure according to the patient's subjective criteria, particularly when the prolapse is axial to the hymenal point. 8 21 22

Based on this scenario, the results of the present study (anterior compartment), showed questionable anatomical cure rates in both groups after one year of follow-up, when considering the cure criterion points of greater POP-Q prolapse below −1 (34.6% and 40%, for USLS and SSLF groups respectively). However, when adopting the hymenal ring as a reference point for healing (Ba ≤ 0), since these patients are asymptomatic, the cure rates were of 88.4% (USLS) and 88% (SSLF).

In the same direction, Barber et al. 23 (2014) compared patients in stages II to IV submitted to SSLF ( *N* = 186) and USLS ( *N* = 188), with 2 years of follow-up, and observed recurrence rates of 13.7% and 15.5% respectively, considering Ba > 0. Then, the results of the present study were similar to the work by Barber et al. 23 , who, after 2 years of follow-up, observed surgical success rates of 59.2% for uterosacral fixation and of 60.5% for sacrospinal fixation, with no difference between the two techniques.

However, Meyer et al. 24 (2020), when reanalyzing data from that study only with patients in stages III and IV, found anterior wall recurrence rates of 16.8% (SSLF) and 17.9% (USLS), and a high rate of patient satisfaction in both groups according to the P-QoL.

The analysis of the apical compartment after the interventions points to the restoration of the anatomy in both groups, with no significant difference. These findings corroborate those of previous studies 2 3 4 15 16 and enable us to demonstrate that both techniques yield satisfactory surgical outcomes. Choosing to ignore less-rigid cure criteria in the treatment of advanced apical prolapse can provide satisfactory anatomical and surgical outcomes for the patient, especially when native tissues are used for pelvic reconstruction.

On the other hand, in the posterior compartment, a better anatomical result was observed in the SSLF group, with a statistical difference, but no difference in terms of the subjective assessment. This was probably due to to posterior deviation of the vaginal axis: the greater the area of dissection of the posterior compartment, the better the anatomical correction. 24

In both groups, there was a significant decrease in the size and width of the genital hiatus after reconstruction of the posterior compartment and perineal body, which is considered a high-impact factor in surgical success and decreased recurrence. Inadequate correction of the genital hiatus can therefore impair the surgical outcome in addition to resulting in recurrence of the prolapse. 25 26

This randomized design, approved by ethics committees and clinical trials plataforms, highlights the methodological rigor and positive impact by offering reliability and resilience of site-specific surgical treatment in advanced POP, in a paradoxical technological appeal and numerous restrictions to synthetic meshes. Synthetic meshes may have their clinical applicability; however, it is increasingly limited due to the high rate of complications. 2 3 4 7 9 21

The recommendation to use classic techniques for POP correction is in line with reference medical societies, such as the IUGA, 9 the American College of Obstetricians and Gynecologists (ACOG), 2 the International Federation of Gynecology and Obstetrics (Fédération Internationale de Gynécologie et d'Obstétrique, FIGO, in French), 3 the American Urogynecologic Society (AUGS), 9 as well as regulatory agencies (such as the FDA). 7 Besides that, the present study emphasizes a subjective criterion of cure, valued by the person most interested in the subject, the patient, without detriment to the anatomical criterion.

On the other hand, sample size and sexual function data (most patients no longer had an active sexual life) may be limitations of the present study which may not compromise the scientific quality. The authors believe that the parity of positive cure outcomes analyzed by different anatomical methods, as well as the application of the P-QoL, reinforces the data and alleviates the limitation of the sample. Besides that, due to the objective being the comparison of cure criteria, not the assessment of the superiority or inferiority of a technique, the sample was sufficient according to the statistical recommendation.

The authors point to the need for longer follow-up of patients (undergoing evaluations to provide data for future studies) with a more robust sample from multiple centers to really assess the potential for POP recurrence between the groups.

Due to the current restrictions on the use of synthetic meshes by specialized medical societies, regulatory agencies and several authors, reconstructive pelvic surgery might be moving toward a return to the classic use of native tissues, even in cases o POP in advanced stages.

## Conclusion

Both techniques (SSLF and USLS) have high success rates, good satisfactory anatomical and subjective outcomes, and a positive impact on the quality of life of patients with apical POP in stages III and IV.

## References

[1] Brown H W Hegde A Huebner M Neels H Barnes H C Marquini G V International urogynecology consultation chapter 1 committee 2: Epidemiology of pelvic organ prolapse: prevalence, incidence, natural history, and service needs Int Urogynecol J Pelvic Floor Dysfunct 2022 33 02 173 187 10.1007/s00192-021-05018-z 34977950

[2] The American College of Obstetricians and Gynecologists (ACOG) Pelvic Organ Prolapse: ACOG Practice Bulletin, Number 214 Obstet Gynecol 2019 134 05 e126 e142 10.1097/AOG.0000000000003519 31651832

[3] FIGO Urogynecology and Pelvic Floor Committee Ugianskiene A Davila G W Su T H FIGO review of statements on use of synthetic mesh for pelvic organ prolapse and stress urinary incontinence Int J Gynaecol Obstet 2019 147 02 147 155 10.1002/ijgo.12932 31353463

[4] This document was developed by the American Urogynecologic Society (AUGS) Guidelines and Statements Committee with assistance of Cassandra L. Carberry, MD, Paul K. Tulikangas, Beri M. Ridgeway, Sarah A. Collins, and Rony A. Adam. This peer-reviewed document reflects clinical and scientific advances as of the date issued and is subject to change. The information should not be construed as dictating an exclusive course of treatment or procedure to be followed. Its content is not intended to be a substitute for professional medical judgment, diagnosis or treatment. The ultimate judgment regarding any specific procedure or treatment is to be made by the physician and patient in light of all circumstances presented by the patient.American Urogynecologic Society Best Practice Statement: Evaluation and Counseling of Patients With Pelvic Organ Prolapse Female Pelvic Med Reconstr Surg 2017 23 05 281 287 10.1097/SPV.0000000000000424Erratum in: Female Pelvic Med Reconstr Surg. 2018 May/Jun;24(3):256. PMID: 28846554 28846554

[5] Margulies R U Rogers M A Morgan D M Outcomes of transvaginal uterosacral ligament suspension: systematic review and metaanalysis Am J Obstet Gynecol 2010 202 02 124 134 10.1016/j.ajog.2009.07.052 20113690

[6] Petri E Ashok K Sacrospinous vaginal fixation–current status Acta Obstet Gynecol Scand 2011 90 05 429 436 10.1111/j.1600-0412.2011.01084.x 21306342

[7] U.S. Food and Drug Administration FDA takes action to protect women's health, orders manufacturers of surgical mesh intended for transvaginal repair of pelvic organ prolapse to stop selling all devices Silver Spring (MD) FDA 2019. Available at:https://www.fda.gov/NewsEvents/Newsroom/PressAnnouncements/ucm636114.htmRetrievedJuly 17, 2019

[8] Pelvic Floor Disorders Network Barber M D Brubaker L Nygaard I Wheeler T L 2nd Schaffer J Chen Z Defining success after surgery for pelvic organ prolapse Obstet Gynecol 2009 114 03 600 609 10.1097/AOG.0b013e3181b2b1ae 19701041 PMC2904469

[9] International Urogynecological Association International Continence Society Haylen B T de Ridder D Freeman R M Swift S E Berghmans B Lee J An International Urogynecological Association (IUGA)/International Continence Society (ICS) joint report on the terminology for female pelvic floor dysfunction Neurourol Urodyn 2010 29 01 4 20 10.1002/nau.20798 19941278

[10] Morley G W DeLancey J O Sacrospinous ligament fixation for eversion of the vagina Am J Obstet Gynecol 1988 158 04 872 881 3364499 10.1016/0002-9378(88)90088-9

[11] Shull B L Bachofen C Coates K W Kuehl T J A transvaginal approach to repair of apical and other associated sites of pelvic organ prolapse with uterosacral ligaments Am J Obstet Gynecol 2000 183 06 1365 1373, discussion 1373–1374 11120498 10.1067/mob.2000.110910

[12] Digesu G A Khullar V Cardozo L Robinson D Salvatore S P-QOL: a validated questionnaire to assess the symptoms and quality of life of women with urogenital prolapse Int Urogynecol J Pelvic Floor Dysfunct 2005 16 03 176 181, discussion 181 10.1007/s00192-004-1225-x 15875234

[13] Haylen B T Freeman R M Swift S E Cosson M Davila G W Deprest J An International Urogynecological Association (IUGA)/International Continence Society (ICS) joint terminology and classification of the complications related directly to the insertion of prostheses (meshes, implants, tapes) & grafts in female pelvic floor surgery Int Urogynecol J Pelvic Floor Dysfunct 2011 22 01 3 15 10.1007/s00192-010-1324-9 21140130

[14] Schulz K F Grimes D A Sample size calculations in randomised trials: mandatory and mystical Lancet 2005 365 9467 1348 1353 10.1016/S0140-6736(05)61034-3 15823387

[15] Eunice Kennedy Shriver National Institute of Child Health and Human Development Pelvic Floor Disorders Network Barber M D Brubaker L Burgio K L Richter H E Nygaard I Weidner A C Comparison of 2 transvaginal surgical approaches and perioperative behavioral therapy for apical vaginal prolapse: the OPTIMAL randomized trial[published erratum appears in JAMA 2015;313:2287]JAMA 2014 311 10 1023 1034 24618964 10.1001/jama.2014.1719 PMC4083455

[16] Larson K A Smith T Berger M B Abernethy M Mead S Fenner D E Long-term patient satisfaction with michigan four-wall sacrospinous ligament suspension for prolapse Obstet Gynecol 2013 122 05 967 975 24104775 10.1097/AOG.0b013e3182a7f0d5 PMC3860104

[17] Maher C Feiner B Baessler K Christmann-Schmid C Haya N Brown J Surgery for women with apical vaginal prolapse Cochrane Database Syst Rev 2016 10 10 CD012376 10.1002/14651858.CD012376 27696355 PMC6457970

[18] Morgan D M Larson K Uterosacral and sacrospinous ligament suspension for restoration of apical vaginal support Clin Obstet Gynecol 2010 53 01 72 85 10.1097/GRF.0b013e3181cf2d51 20142645

[19] Diwadkar G B Barber M D Feiner B Maher C Jelovsek J E Complication and reoperation rates after apical vaginal prolapse surgical repair: a systematic review Obstet Gynecol 2009 113(2 Pt 1):367 373 10.1097/AOG.0b013e318195888dErratum in: Obstet Gynecol. 2009 Jun;113(6):1377. PMID: 19155908 19155908

[20] Weber A M Walters M D Piedmonte M R Ballard L A Anterior colporrhaphy: a randomized trial of three surgical techniques Am J Obstet Gynecol 2001 185 06 1299 1304, discussion 1304–1306 10.1067/mob.2001.119081 11744900

[21] Samuelsson E C Victor F T Tibblin G Svärdsudd K F Signs of genital prolapse in a Swedish population of women 20 to 59 years of age and possible related factors Am J Obstet Gynecol 1999 180(2 Pt 1):299 305 10.1016/s0002-9378(99)70203-6 9988790

[22] Pelvic Floor Disorders Network Nygaard I Barber M D Burgio K L Kenton K Meikle S Schaffer J Prevalence of symptomatic pelvic floor disorders in US women JAMA 2008 300 11 1311 1316 10.1001/jama.300.11.1311 18799443 PMC2918416

[23] Eunice Kennedy Shriver National Institute of Child Health and Human Development Pelvic Floor Disorders Network Barber M D Brubaker L Burgio K L Richter H E Nygaard I Weidner A C Comparison of 2 transvaginal surgical approaches and perioperative behavioral therapy for apical vaginal prolapse: the OPTIMAL randomized trial JAMA 2014 311 10 1023 1034 10.1001/jama.2014.1719Erratum in: JAMA. 2015 Jun 9;313(22):2287. PMID: 24618964; PMCID: PMC4083455 24618964 PMC4083455

[24] NICHD Pelvic Floor Disorders Network and the National Institutes of Health Office of Research on Women's Health Meyer I Whitworth R E Lukacz E S Smith A L Sung V W Visco A G Outcomes of native tissue transvaginal apical approaches in women with advanced pelvic organ prolapse and stress urinary incontinence Int Urogynecol J Pelvic Floor Dysfunct 2020 31 10 2155 2164 10.1007/s00192-020-04271-y PMC7483223 32146521

[25] Delancey J O Hurd W W Size of the urogenital hiatus in the levator ani muscles in normal women and women with pelvic organ prolapse Obstet Gynecol 1998 91 03 364 368 10.1016/s0029-7844(97)00682-0 9491861

[26] Vaughan M H Siddiqui N Y Newcomb L K Weidner A C Kawasaki A Visco A G Bradley M S Surgical Alteration of Genital Hiatus Size and Anatomic Failure After Vaginal Vault Suspension Obstet Gynecol 2018 131 06 1137 1144 10.1097/AOG.0000000000002593 29742664

